# Perioperative outcomes of robotic-assisted vs. conventional laparoscopy for colorectal cancer resection: a systematic review and meta-analysis

**DOI:** 10.3389/fsurg.2026.1723076

**Published:** 2026-02-25

**Authors:** Alaa R. Al-Ihribat, Ibrahim Moqbel, Ahmed Oun, Ahmed Mahmoud Ahmed Mekky, Mohamed Youssef Abdou Youssef, Mohamed Fawzy Abdelkader Youssef, Hamza Khelifa, Fatima Mohammed Elawad Sanhour, Ashraf Abdelmonem Elsayed

**Affiliations:** 1 College of Medicine and Health Sciences, Palestine Polytechnic University, Hebron, Palestine; 2 Faculty of Medicine, Cairo University, Cairo, Egypt; 3 Tanta University Hospital, Tanta, Egypt; 4 Faculty of Medicine, Aswan University, Aswan, Egypt; 5 Faculty of Medicine, Zagazig University, Zagazig, Egypt; 6 Alexandria University Faculty of Medicine, Alexandria, Egypt; 7 Faculty of Medicine, University of Oran 1 Ahmed Ben Bella, Oran, Algeria; 8 University of Khartoum, Khartoum, Sudan

**Keywords:** colorectal cancer, laparoscopic surgery, meta-analysis, postoperative outcomes, robotic surgery, systematic review

## Abstract

**Background:**

Colorectal cancer is a major global health concern that requires successful surgical treatments. While robotic-assisted surgery (RAS) provides prospective improvements, laparoscopic surgery has proven to yield better results than open surgeries.

**Methods:**

From 2018 to December 2024, PubMed, Scopus, and Web of Science were used to perform a systematic review and meta-analysis of cohort studies and randomized controlled trials (RCTs). Studies comparing RAS and conventional laparoscopic surgery were included. The primary outcomes assessed were length of hospital stay, conversion to open surgery, postoperative complications, and operating time. Using Comprehensive Meta-Analysis software, statistical analysis was performed, including subgroup analyses by anatomical site (colon, rectum, colorectal). Sensitivity analyses and heterogeneity were conducted.

**Results:**

21 studies involving over 70,000 patients were included. The meta-analysis demonstrated significantly longer operative times with RAS (MD = 0.161–1.049, *p* < 0.001). RAS was linked to a significantly lower chance of re-operative rates (RR = 0.549, *p* = 0.023) and a significantly lower risk of conversion to open surgery (RR = 0.412–0.592, *p* < 0.001). RAS decreased problems in the colorectal group (RR = 0.867, *p* = 0.023), but overall rectum group complication rates were comparable. Hospital stays were shorter after robotic-assisted surgery (MD = −0.284 to −0.755, *p* = 0.001).

**Conclusion:**

When compared to CLS, RAS has the advantage of lowering conversion and re-operation rates, albeit at the expense of higher operating time. CLS led to shorter hospital stays, but in some circumstances, the complication rates were on level with or lower than those of RAS. According to these results, RAS might be useful in some surgical situations and patient demographics.

**Systematic Review Registration:**

https://www.crd.york.ac.uk/PROSPERO/view/614084, PROSPERO CRD42024614084.

## 1 Introduction

Colorectal cancer—the third most common cancer and the second most common cause of cancer related deaths—represents a huge social and economic burden (1, 2). For women, it represents the 2nd most common cancer, for men it represents the 3rd most common cancer (3). The incidence increased among people younger than 50 years (4). Risk factors include male gender, old age, inflammatory bowel disease, family history in a first-degree relative, high BMI, smoking, and red meat intake (3, 5). Its treatment differs according to stage and location, but surgery remains the mainstay of treatment. Surgical approach differs according to location and can be either open, laparoscopic or robotic.

Jacobs et al. (6) introduced laparoscopic surgery for colonic resection in 1991. Laparoscopic surgery overcomes many shortcomings of open surgery and offers many advantages: smaller incision, better cosmetically, less hospital stay duration and less postoperative morbidity and mortality (7, 8). The two-dimensional visualization, decreased ergonomics and tremor effect represent disadvantages (9).

Weber et al. (10) introduced robotic surgery for colonic resection in 2002. Robotic surgery overcomes many shortcomings of laparoscopic surgery and offers many advantages: three-dimensional visualization, better ergonomics, reducing tremor effect and stable camera (11–13). The high cost compared to laparoscopic surgery represents a disadvantage (14).

Many studies have compared the outcomes of both the laparoscopic and robotic surgery for colorectal cancer; the superiority of either of them remains an unsolved question. Many of the studies used retrospective study design and had small sample size and this necessitates careful usage of the results of individual studies in clinical practice. The aim of this systematic review was to compare between laparoscopic and robotic surgery for colorectal cancer in terms of postoperative outcomes.

Although several meta-analyses have compared robotic-assisted and conventional laparoscopic surgery for colorectal cancer, most were conducted prior to 2018 and therefore did not include the substantial increase in robotic utilization and contemporary high-volume datasets published in recent years. Our review incorporates the most up-to-date evidence (2018–2024), includes one of the largest pooled patient populations to date, and provides a more granular comparison through separate subgroup analyses for colon, rectal, and colorectal procedures. These stratified analyses, combined with updated perioperative outcomes and broader geographic representation, allow for a more accurate appraisal of current surgical practice and address gaps not fully explored in previous reviews.

## 2 Method

This systematic review and meta-analysis was conducted following the Cochrane Handbook for Systematic Reviews of Interventions (15) and reported in accordance with PRISMA guidelines (16) with registration in PROSPERO (CRD42024614084). Studies were eligible if they were open-access, English-language randomized controlled trials or cohort studies published between 2018 and December 2024, comparing robotic-assisted and conventional laparoscopic resection in patients with operable colorectal cancer. Excluded studies included systematic reviews, meta-analyses, literature reviews, non-open access articles, and those lacking preoperative or postoperative outcome data. Two independent reviewers screened titles and abstracts, resolving discrepancies by consensus or with a third reviewer's input.

### 2.1 Search strategy and information sources

A comprehensive literature search was performed across PubMed, Cochrane Library, Scopus, and Web of Science to ensure the inclusion of all pertinent studies from 2018 to December 2024. The search strategy integrated both Medical Subject Headings (MeSH) and free-text terms, utilizing Boolean operators (AND, OR) to combine key terms such as “robotic surgical procedure,” “robot assisted surgery,” “Colorectal Neoplasm,” “Colorectal Cancer,” and “Conventional Laparoscopy.” This robust search approach was designed to capture all relevant literature within the specified timeframe and ensure that no significant studies were overlooked.

The literature search was restricted to studies published from 2018 onward. This timeframe was selected to ensure inclusion of contemporary robotic systems and current perioperative practices, as earlier studies predominantly evaluated outdated robotic platforms and were conducted before widespread implementation of ERAS protocols. Limiting the search to 2018–2024 therefore reduces clinical heterogeneity related to technological evolution and standardization of care, and provides a more accurate reflection of modern colorectal surgical practice.

### 2.2 Data extraction and management

Data extraction was carried out independently by four investigators using a standardized electronic form to guarantee consistency and reliability. Key details extracted from each study included study identification, study period, country and setting, sample size, and patient characteristics such as age, sex, body mass index, and ASA score, tumor stage details. Additionally, we recorded a range of clinical outcomes comprising intraoperative measures (operative time, blood loss, conversion rate to open surgery, length of hospital stay, overall complication rate), pathological outcomes (proximal and distal resection margins, number of lymph nodes harvested), and postoperative recovery parameters (Time to first flatus, time to first solid diet, 30-day readmission, reoperation rate, and mortality). Definitions of specific postoperative outcomes were standardized across studies:

Bleeding was defined as any postoperative hemorrhage requiring transfusion, reoperation, or documented hemoglobin drop as reported by each study.
Infection included superficial or deep surgical site infection, intra-abdominal abscess, or documented postoperative infectious complications.
Ileus was defined as delayed return of bowel function requiring nasogastric decompression, prolonged fasting, or radiologic confirmation.

Postoperative complications were extracted as reported in each study. Whenever possible, we recorded complications according to the Clavien–Dindo classification. Eight of the included studies explicitly used the Clavien–Dindo system, including Farah et al., DeBakey et al., Wei et al., and others. For studies that did not report complications with Clavien–Dindo grading, we extracted each complication exactly as defined by the original authors and grouped them under standardized categories (e.g., bleeding, infection, ileus) to allow consistent comparison across studies. Any discrepancies encountered during the extraction process were resolved by re-verification of the source material and discussion with a senior investigator.

Two randomized trials by Feng et al. [references (31, 33)] were included in this review. Although both originate from the same research group, they represent independent studies with different sample sizes, recruitment periods, and outcome focuses. Full-text comparison confirmed that no patient overlap existed between the two datasets.

### 2.3 Quality assessment

**Figure 1:**
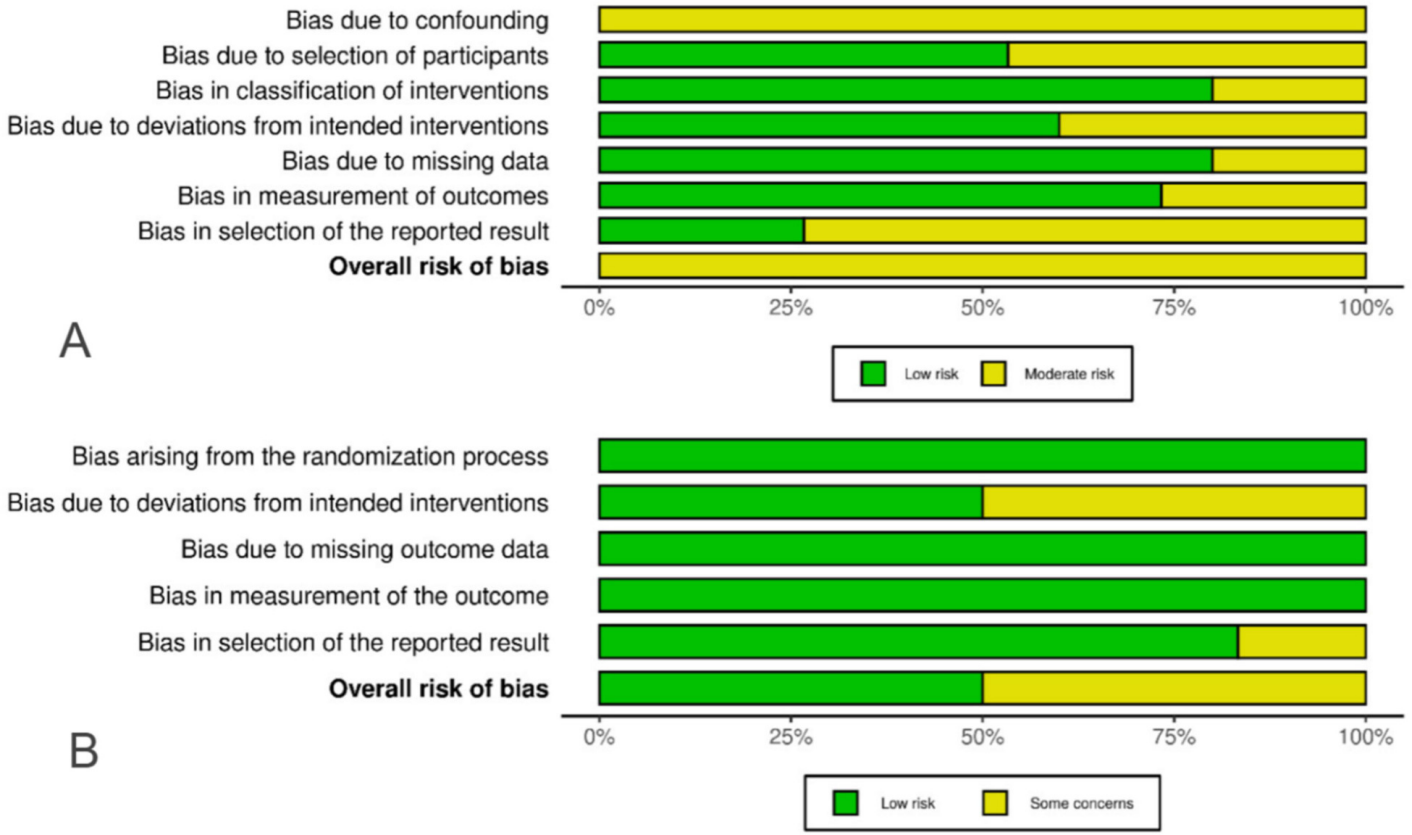
Assessment of risk of bias in the included studies. **(A)** ROBINS-1 evaluation of observational studies. The panel presents a schematic representation of risks (low = green, moderate = yellow) for specific types of biases of each study in the review. **(B)** ROB-2 evaluation of RCTs. The panel presents a schematic representation of risks (low = green, Some concerns = yellow) for specific types of biases of each study in the review.

Two independent reviewers conducted a risk of bias assessment for the studies included in the analysis. For randomized controlled trials (RCTs), the Cochrane Risk of Bias 2 (ROB2) tool was utilized to assess domains including the randomization process, deviations from intended interventions, missing outcome data, measurement of outcomes, and selection of reported results. Each domain was carefully evaluated, and a risk decision (low, some concerns, or high) was made with detailed justifications provided. For non-randomized studies of interventions, the Risk Of Bias In Non-randomized Studies—of Interventions (ROBINS-I) assessment tool was applied. This tool assesses various factors, including randomization, allocation concealment, blinding, selective reporting, and additional sources of bias. Based on this comprehensive assessment, the included studies were categorized as having a “low risk” of bias, “high risk” of bias, or 'some concerns' (Figure 1). Any disagreements were resolved through discussion, and a third author was consulted when necessary.

### 2.4 Statistical analysis

Statistical analysis was performed using Review Manager (RevMan) version 5.4 (The Cochrane Collaboration, 2020). A random-effects model was applied to account for potential heterogeneity across studies. Continuous outcomes were analyzed using mean differences (MD) with corresponding 95% confidence intervals (CI), while dichotomous (categorical) outcomes were reported as risk ratios (RR) with 95% CI. Statistical significance was defined as a *p*-value less than 0.05. Heterogeneity among studies was assessed using the *I*^2^ statistic, with values of 25%, 50%, and 75% representing low, moderate, and high heterogeneity, respectively. Subgroup analyses were performed based on the anatomical site of surgery (colon, rectum, or colorectal) to enhance interpretability. Forest plots were generated to visually represent pooled effect estimates for each outcome.

### 2.5 Heterogeneity

A visual inspection of the final forest plots and an assessment by I-square and Chi-Square tests (Cochran's *Q* test) were conducted to identify the degree of heterogeneity. In case of significant heterogeneity (Chi-Square *P* < 0.1).

## 3 Results

### 3.1 Search strategy and study selection

**Figure 2:**
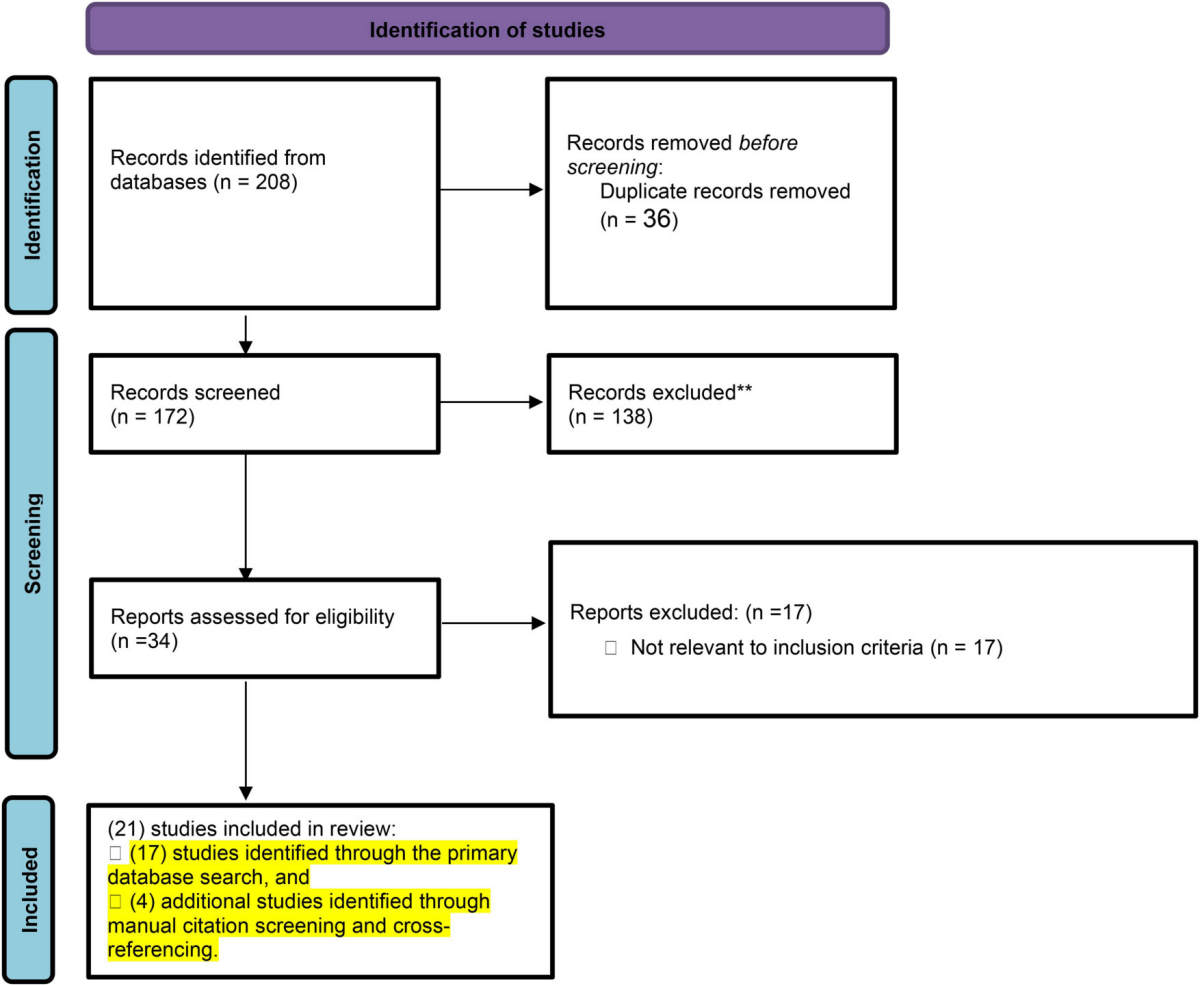
The PRISMA flow diagram.

We identified 208 records through database searches (PubMed, Scopus, Web of Science). After removing 36 duplicates, we screened 172 titles and abstracts against the inclusion criteria, excluding 138. Thirteen additional studies were excluded during the full-text review for abstracts, reviews, and not relevant studies. Ultimately, 21 studies met eligibility and were included in the meta-analysis [references (17–37)] (Figure 2).

### 3.2 Characteristics of included studies

The 21 included studies comprised six randomized controlled trials (RCTs) and 15 observational cohort studies, published between 2018 and 2024. These studies collectively enrolled over 70,000 patients across multiple countries, including the United States, Canada, the United Kingdom, Germany, Brazil, India, China, and Australia. Interventions varied by robotic platform, operative technique, and surgeon experience, while all control groups underwent conventional laparoscopic colorectal surgery [Table 1].

**Table 1:** Baseline patients and summary characteristics.

ID	Tumor site	Surgical approach	Sample size	Age	Gender (male %)	BMI
Cawich et al. (24)	Colon	FreeHand® robot-assisted laparoscopic colectomy	8	59.9 ± 6.90	63%	NA
		Conventional laparoscopic colectomy	15	57.9 ± 8.43	53%	NA
Chung et al. (26)	Colorectal	Robot-assisted surgery	2,048	80.7 ± 0.09	48.90%	NA
		Laparoscopic surgery	2,048	80.8 ± 0.09	48.90%	NA
Farah et al. (27)	Colon (Right colectomy)	Robot-assisted surgery	2,352	NA	47%	NA
		Laparoscopic surgery	4,704	NA	46.50%	NA
	Colon (Left colectomy)	Robot-assisted surgery	3,123	NA	52.30%	NA
		Laparoscopic surgery	6,246	NA	52.30%	NA
	Rectum (Low anterior resection)	Robot-assisted surgery	4,854	NA	60.80%	NA
		Laparoscopic surgery	4,854	NA	59.70%	NA
Kim et al. (29)	Colon	Single-incision robotic colectomy	43	58.8 ± 7.7	27.90%	3.00%
		conventional multiport laparoscopic colectomy	97	70.6 ± 7.7	64.90%	7.70%
Park et al. (21)	Rectum	Robot-assisted surgery	151	65.5 ± 11.4	64.20%	23.9 ± 3.3
		Laparoscopic surgery	144	67.2 ± 10.1	68.80%	23.5 ± 2.8
Sterk et al. (37)	Colon	Robot-assisted surgery	1,105	71 ± 10.91	54.10%	26.13 ± 3.90
		Laparoscopic surgery	14,901	70.67 ± 10.94	53.30%	26.10 ± 4.24
Feng et al. (31)	Rectum	Robot-assisted surgery	586	59.1 ± 11.0	60.80%	23.5 ± 3.2
		Laparoscopic surgery	585	60.7 ± 9.8	60.50%	23.5 ± 3.0
Feng et al. (33)	Rectum	Robot-assisted surgery	174	58.2 ± 9.6	62.10%	NA
		Laparoscopic surgery	173	59.5 ± 10.9	65.30%	NA
Hettiarachchi et al. (23)	Colorectal	Robot-assisted surgery	74	NA	56.80%	27.8 ± 4.3
		Conventional laparoscopic surgery	110	NA	67.30%	27.8 ± 4.3
Wei et al. (22)	Colorectal	Robotic reduced-port surgery	17	63.4 ± 12.0	41.17%	24.1 ± 2.9
		Laparoscopic surgery	49	66.2 ± 12.5	38.78%	23.6 ± 3.4
Hu et al. (35)	Colorectal	Robot-assisted surgery	271	NA	59.80%	NA
		Laparoscopic surgery	309	NA	56.30%	NA
Oshio et al. (28)	Rectum	Robot-assisted surgery	36	NA	63.90%	22.5
		Laparoscopic surgery	95	NA	63.20%	23.8
Palomba et al. (32)	Colorectal	Robot-assisted surgery	35	NA	59.38%	NA
		Laparoscopic surgery	51	NA	60.78%	NA
Galata et al. (18)	Rectum	Robot-assisted surgery	18	60 ± 11.8	55.60%	26 ± 4
		Conventional laparoscopic surgery	33	62.3 ± 13.7	63.60%	27.4 ± 5.5
Debakey et al. (34)	Rectum	Robot-assisted surgery	21	51.45 ± 9.262	42.40%	NA
		Laparoscopic surgery	24	50.15 ± 7.191	54.20%	NA
Park et al. (21)	Colon	Robot-assisted surgery	35	62.8 ± 10.5	40%	24.4 ± 2.5
		Laparoscopic surgery	35	66.5 ± 11.4	45.70%	23.8 ± 2.7
Fransgaard et al. (19)	Colon	Robot-assisted surgery	511	NA	45.40%	NA
		Laparoscopic surgery	8,104	NA	49.20%	NA
	Rectum	Robot-assisted surgery	760	NA	65.50%	NA
		Laparoscopic surgery	3,934	NA	61.50%	NA
Pinar et al. (25)	Colon	Robot-assisted surgery	311	NA	52%	NA
		Conventional laparoscopic surgery	5,647	NA	50.30%	NA
	Rectum	Robot-assisted surgery	449	NA	62.80%	NA
		Conventional laparoscopic surgery	2,757	NA	61.10%	NA
Kim et al. (29)	Colon	Robot-assisted surgery	66	60.4 ± 9.7	77.30%	24.1 ± 3.3
		Laparoscopic surgery	73	59.7 ± 11.7	71.20%	23.6 ± 3.0
Gorgun et al. (30)	Rectum	Robot-assisted surgery	29	58.8 ± 10.7	75.90%	34.9 ± 7.2
		Laparoscopic surgery	27	60.3 ± 9.8	59.30%	35.2 ± 5
Tam et al. (17)	Colon	Robot-assisted surgery	244	NA	NA	NA
		Laparoscopic surgery	1,142	NA	NA	NA
	Rectum	Robot-assisted surgery	165	NA	NA	NA
		Laparoscopic surgery	369	NA	NA	NA

Across studies, patient mean age ranged from 50.2 to 80.8 years, with a male predominance (57.8%). Reported mean BMIs varied from 22.5 to 35.2. When available, ASA physical status distribution was as follows: ASA I (31%), ASA II (45%), and ASA III–IV (22%). Tumor staging was diverse: 20.6% stage I, 27.0% stage II, 38.9% stage III, and 16.8% stage IV (Table 1).

### 3.3 Risk of bias assessment

RCTs were evaluated using the Cochrane RoB 2.0 tool: three trials were rated low risk, while three exhibited “some concerns” due to deviations from intended interventions. The observational studies were assessed with ROBINS-I and uniformly demonstrated moderate risk of bias (Figure 1) (Supplementary Figures 12S, 13S).

### 3.4 Outcomes

To reduce heterogeneity and enhance the robustness of the results, subgroup analyses were performed based on the anatomical site of the surgical procedure, categorizing studies into colon, rectum, and colorectal groups.

#### 3.4.1 Operative time

**Figure 3:**
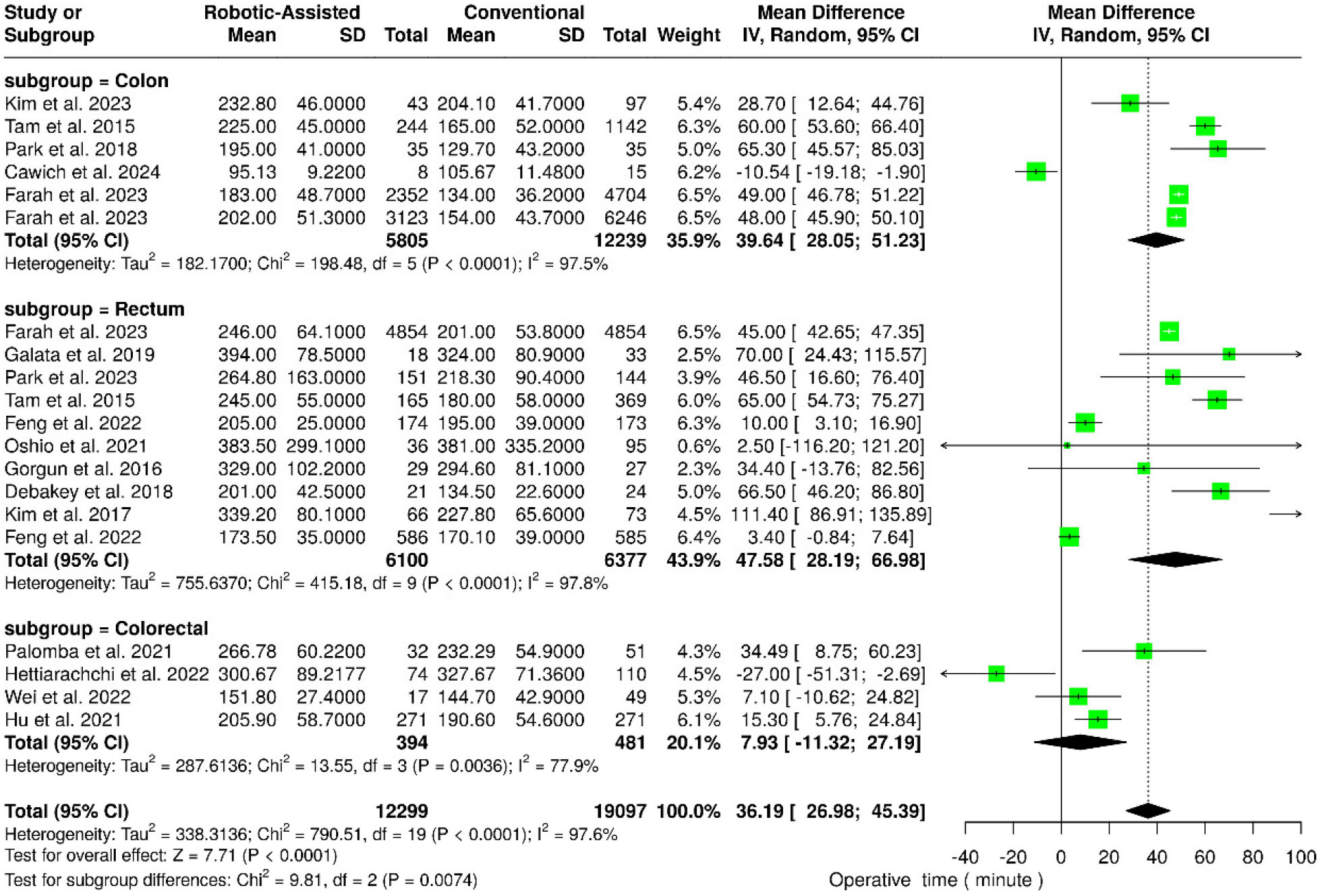
Forest plots illustrate the analysis of operative time.

Subgroup analysis of six studies in the colon subgroup revealed that robotic-assisted surgery took significantly longer operative time compared to conventional laparoscopic surgery [Mean Difference (MD): 39.64 min; 95% CI: 28.05–51.23; *p* < 0.001; *I*^2^ = 97.5%] (Figure 3). Similarly, in the rectum subgroup (10 studies), robotic-assisted operations lasted significantly longer operative times (MD: 47.58 min; 95% CI: 28.19–66.98; *p* < 0.001; *I*^2^ = 97.8%). In contrast, the colorectal subgroup (4 studies) no statistically significant difference was observed in the operative time between the two approaches (MD: 7.93 min; 95% CI: −11.16 to 27.29; *p* = 0.42; *I*^2^ = high).

#### 3.4.2 Conversion to open surgery

**Figure 4:**
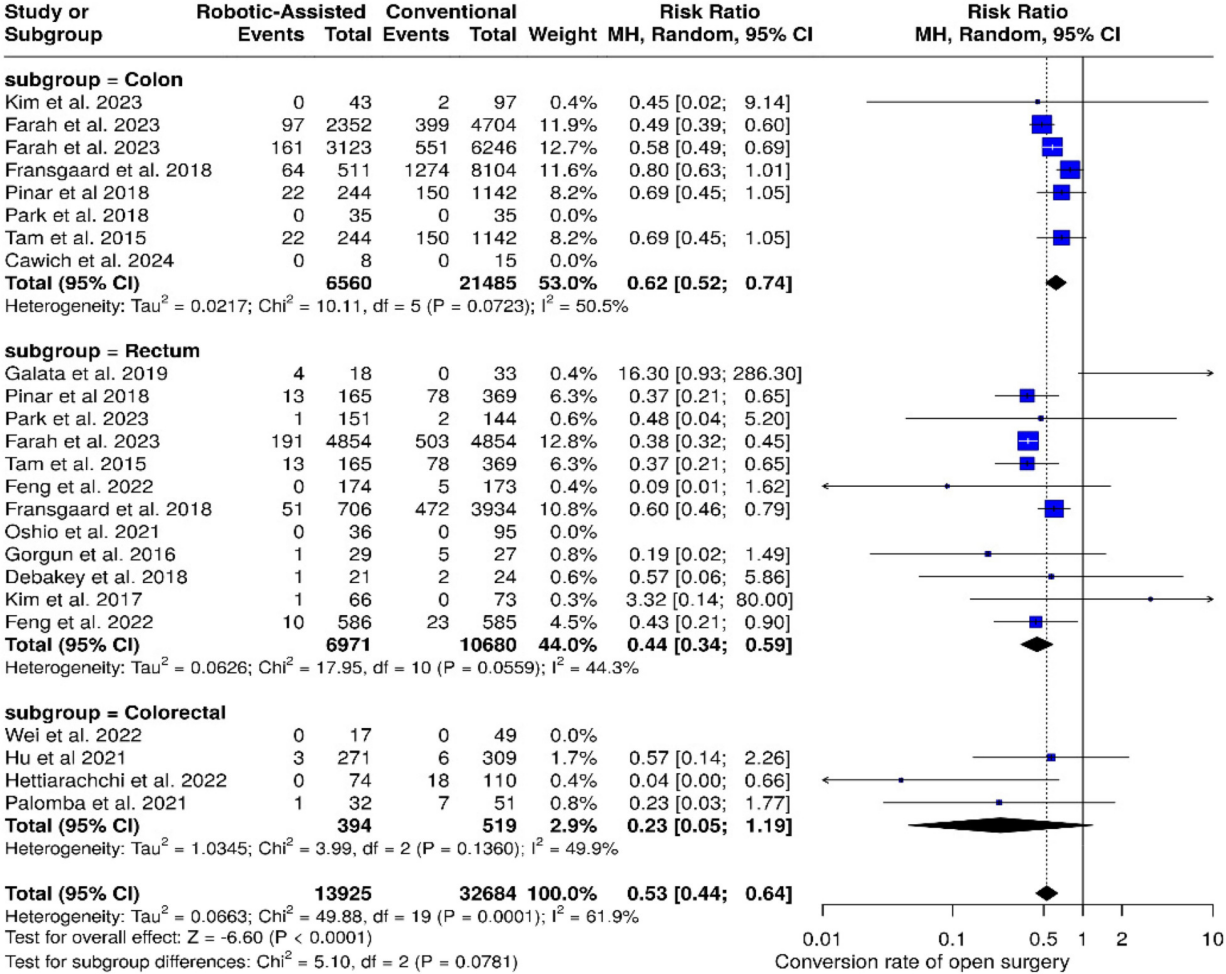
Forest plots illustrate the analysis of conversion to open surgery.

In the colon subgroup (8 studies), robotic-assisted surgery had significantly lower conversion rates compared to laparoscopy [Risk Ratio (RR): 0.62; 95% CI: 0.52–0.74; *p* < 0.001; *I*^2^ = 50.5%]. The rectum subgroup (12 studies) also had a corresponding benefit for robotic surgery (RR: 0.44; 95% CI: 0.34–0.59; *p* < 0.001; *I*^2^ = 44.3%). In the subgroup for colorectal (4 studies), the trend existed for robotic surgery but the result was not significant (RR: 0.23; 95% CI: 0.05–1.19; *p* = 0.079; *I*^2^ = 61.9%) (Figure 4).

#### 3.4.3 Overall complications

**Figure 5:**
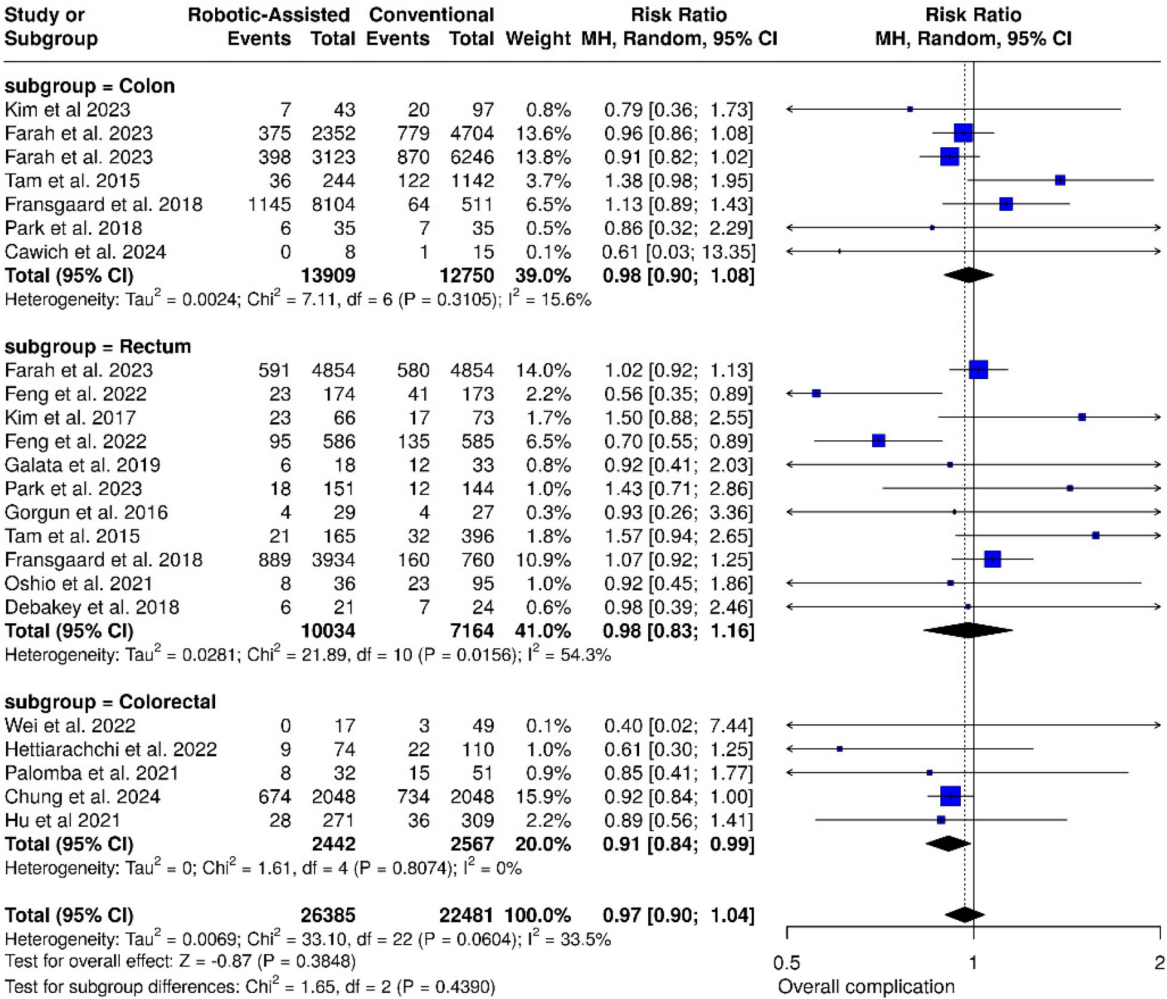
Forest plots illustrate the analysis of overall complication rate.

In 7 studies of the colon subgroup, the two procedures were not significantly different regarding postoperative complication rates (RR: 0.98; 95% CI: 0.90–1.08; *p* = 0.079; *I*^2^ = 15.6%). in rectum subgroup (11 studies) no significant difference was found (RR: 0.98; 95% CI: 0.83–1.16; *p* = 0.81; *I*^2^ = 54.3%). However, the colorectal subgroup (4 studies) showed significantly fewer complications in the robotic-assisted group (RR: 0.91; 95% CI: 0.84–0.99; *p* = 0.026; *I*^2^ = 0%) (Figure 5).

#### 3.4.4 Length of hospital stay

Robot -assisted surgery was found to be linked to shorter hospital stay across all subgroups. In the colon subgroup (8 studies), the reduction was statistically significant (MD: −0.59 days; 95% CI: −0.78 to −0.41; *p* < 0.001; *I*^2^ = 66.4%). The rectum subgroup (10 studies) also demonstrated a shorter stay for robotic procedures (MD: −0.68 days; 95% CI: −1.15 to −0.20; *p* = 0.005; *I*^2^ = 93.2%). In the colorectal subgroup (3 studies), robotic surgery led to significantly greater reduction in hospital stay (MD: −3.68 days; 95% CI: −5.28 to −2.07; *p* < 0.001; *I*^2^ = 92.6%) (Supplementary Figure 6S).

#### 3.4.5 Readmission rate

There was no significant variation within the colon subgroup (4 studies) between robotic and laparoscopic approaches (RR: 1.01; 95% CI: 0.91–1.13; *p* = 0.80; *I*^2^ = 0%). Notably, in the rectum subgroup (8 studies), robotic-assisted surgery was associated with a slightly higher readmission rate (RR: 1.13; 95% CI: 1.00–1.26; *p* = 0.042; *I*^2^ = 20.2%). The colorectal subgroup had a single study included, which likewise did not show any statistically significant difference (RR: 0.22; 95% CI: 0.01–3.67; *p* = 0.026; *I*^2^ = 2.6%) (Supplementary Figure 8S).

#### 3.4.6 Reoperation rate

Eight studies participated in reoperation rate meta-analysis in colon, rectum, and colorectal subgroups. In the rectum subgroup (6 studies), robotic surgery was statistically significantly associated with lower reoperation rates compared to conventional laparoscopy [Risk Ratio (RR): 0.55; 95% CI: 0.34–0.91; *p* = 0.022; *I*^2^ = 0%]. This indicates high consistency between studies. On the other hand, no such difference was found in the colon subgroup (2 studies; RR: 1.00; 95% CI: 0.07–15.36) or colorectal subgroup (1 study; RR: 0.80; 95% CI: 0.08–8.43). The total pooled analysis showed a statistically significant lower risk of reoperation with robotic surgery (RR: 0.57; 95% CI: 0.35–0.92; *p* = 0.0223; *I*^2^ = 0%), and no subgroup difference (*p* = 0.8809) (Supplementary Figure 9S).

#### 3.4.7 Intraoperative blood loss

The colon subgroup (3 studies) did not show any difference between groups in blood loss (MD: −9.70 mL; 95% CI: −21.50 to 2.11; *p* = 0.11; *I*^2^ = 0%). However, in the rectum subgroup (8 studies), robotic-assisted surgery had less blood loss significantly (MD: −38.84 mL; 95% CI: −56.55 to −21.12; *p* < 0.001; *I*^2^ = 97.9%). In the colorectal subgroup (2 studies), robotic surgery was associated with more blood loss, although not statistically (MD: 19.67 mL; 95% CI: −26.16 to 65.51; *p* = 0.40; *I*^2^ = 96.7%) (Supplementary Figure 3S).

#### 3.4.8 Bleeding rate

No statistically significant difference in bleeding rate was found in the colon subgroup (4 studies) (RR: 0.96; 95% CI: 0.84–1.09; *p* = 0.49; *I*^2^ = 5.1%) or the rectum subgroup (5 studies) (RR: 0.84; 95% CI: 0.67–1.06; *p* = 0.14; *I*^2^ = 0%). In contrast, the colorectal subgroup (3 studies) showed significantly fewer bleeding events with robotic surgery (RR: 0.55; 95% CI: 0.46–0.65; *p* < 0.001; *I*^2^ = 69.6%) (Supplementary Figure 2S).

#### 3.4.9 Lymph node harvest

19 studies evaluated lymph node yield in the colon, rectum, and colorectal subgroups. In the colon subgroup (6 studies), robotic-assisted surgery was significantly greater for lymph node harvest compared to conventional laparoscopy [Mean Difference (MD): 0.85; 95% CI: 0.03–1.67; *p* = 0.04; *I*^2^ = 58.4%]. In the rectum subgroup (10 studies), the advantage was even more pronounced, with robotic-assisted surgery yielding a significantly greater number of lymph nodes (MD: 1.16; 95% CI: 0.12–2.20; *p* < 0.001; *I*^2^ = 90.6%). However, in the colorectal subgroup (2 studies), no statistically significant difference was observed (MD: −1.21; 95% CI: −4.79 to 2.36; *p* = 0.51; *I*^2^ = 46.4%). The overall pooled analysis showed a modest but statistically significant increase in lymph node yield with robotic-assisted surgery (MD: 0.76; 95% CI: 0.12–1.39; *p* = 0.02; *I*^2^ = 85.2%) (Supplementary Figure 7S).

While lymph node yield was consistently reported across most of the included studies and therefore could be meta-analyzed, other oncologic quality parameters—such as total mesorectal excision (TME) quality, circumferential resection margin involvement, distal resection margins, local recurrence, disease-free survival, and overall survival—were not uniformly or sufficiently reported. The heterogeneity and limited availability of these oncologic outcomes across studies prevented quantitative synthesis, and thus only lymph node harvest could be reliably included in the pooled analysis.

#### 3.4.10 Anastomotic leak

There were no significant differences in anastomotic leak rates across any subgroup. Colon subgroup (5 studies): RR: 1.15; 95% CI: 0.93–1.44; *p* = 0.20; *I*^2^ = 0%. Rectum subgroup (10 studies): RR: 1.22; 95% CI: 1.01–1.48; *p* = 0.043; *I*^2^ = 0%. Colorectal subgroup (3 studies): RR: 0.97; 95% CI: 0.38–2.51; *p* = 0.955; *I*^2^ = 0% (Supplementary Figure 1S).

#### 3.4.11 Infection rate

Robotic-assisted surgery was associated with a lower infection rate in the colon subgroup (3 studies) (RR: 0.49; 95% CI: 0.28–0.85; *p* = 0.01; *I*^2^ = 0%). No significant difference was observed in the rectum subgroup (7 studies) (RR: 0.99; 95% CI: 0.67–1.48; *p* = 0.85; *I*^2^ = 0%) or colorectal subgroup (2 studies) (RR: 0.61; 95% CI: 0.45–0.83; *p* < 0.001; *I*^2^ = 0%) (Supplementary Figure 5S).

#### 3.4.12 Ileus

Robotic surgery was associated with significantly lower ileus rates in the colon subgroup (3 studies) (RR: 0.85; 95% CI: 0.74–0.97; *p* = 0.016; *I*^2^ = 25.9%). No significant differences were observed in the rectum (9 studies) or colorectal (1 study) subgroups (Supplementary Figure 4S).

#### 3.4.13 Time to first flatus and solid diet

Time to first flatus was significantly shorter in the colon and rectum subgroups for robotic surgery, though heterogeneity was high. Results for time to solid diet were inconclusive, with no statistically significant differences found (Supplementary Figure 10S, 11S).

## 4 Discussion

According to the latest update of cancer statistics, based on the American Cancer Society 2024 (38), Colorectal Cancer (CRC) was the fourth-leading cause of cancer death in both men and women younger than 50 years in the late-1990s. However, it is now the first in men and second in women. The rising in CRC incidence requested additional exploration of surgical techniques that optimize patient outcomes, as colorectal resection considered the gold standard treatment for CRC (39). We conducted a systematic review and meta-analysis of 21 RCTs and Cohort studies that aimed to compare Robotic-Assisted surgery (RAS) with Conventional Laparoscopic Surgery (CLS) for CRC, focusing on various outcomes. Our findings indicate that, despite its association with longer operative time (OT), RAS offers significant advantages in terms of reduced conversion rates to open surgery, as well as re-operative rates, and lower complication rates in certain contexts.

To reduce heterogeneity and enhance the robustness of the results, subgroup analyses were performed based on the anatomical site of the surgical procedure, categorizing studies into colon, rectum, and colorectal groups. RAS showed a statistically significant increase in OT compared to CLS among all anatomical sites examined, this finding aligns with previous meta-analysis of 169,236 patients by Ting-Ng et al. (40) which also reported a longer OT of RAS, also highlighted the benefits in reducing conversion rates and overall complications. Similarly, a systematic review of published randomized controlled trials (RCT) for 3,307 patients by Huang et al. (41) underscore that while RAS may be time-consuming, it can lead to better overall patient outcomes, as it is associated with lower conversion rates and shorter hospital stays.

In term of overall complications, our review demonstrated no significant difference in the incidence of overall complications between RAS and CLS in the rectum group. However, the colorectal group showed a significant decrease in complications associated with RAS, which was in line to other meta-analysis. For instance, trustfully et al. (42), reported a significantly lower incidence of overall postoperative complications in patients undergoing colonic resections for Cancer and Benign Diseases by RAS. However, Xiong et al. (43) demonstrated no statistical significance between RAS and CLS in terms of postoperative morbidity and mortality for patients who underwent total mesorectal excision for rectal cancer. These findings underscore the superiority of RAS in colon-related surgeries, with the presence of some heterogeneity among the included studies and variations in surgical techniques and patient populations. Furthermore, in this study, we analyze specific postoperative complications, including infection and ileus, our analysis showed a decreased rate of these complications following RAS, which further supports the notion that robotic-assisted surgery may offer distinct advantages over conventional laparoscopic approaches in minimizing specific postoperative complications. However, different studies only indicate a decreased overall complication rates following RAS (44).

Our pooled analysis for rectum group of readmission and reoperation rates showed significant increase in reoperation rates associated with conventional surgery, as this match with Goldstone et al. (45) who studied the outcomes comparison of robotic-assisted vs. laparoscopic and open surgery for patients undergoing rectal cancer resection, which suggested that RAS may reduce the need for reoperations due to its precision and enhanced visualization capabilities.

Despite subgroup analyses that performed based on the anatomical site to reduce heterogeneity, the observed heterogeneity can be attributed to several factors, including variations in surgeon experiences, patient demographics, and the specific methodology in some analyzed studies. For instance, Farah et al. (27) mentioned that that patient selection and the procedure performed (right colectomy, left colectomy, and low anterior resection) may introduce some heterogeneity, in their analysis, outcomes varied depending on which type of colorectal resection was performed, with robotic surgery showing advantages in some procedures (like right colectomy and left colectomy) but not in low anterior resection, which may be related to anatomical differences and the technical challenges unique to rectal surgery. Additionally, Feng et al. (33), studied the difference between the two surgical approaches in 11 Chinese centers, however, the lack of standardized perioperative protocols across these centers could also contribute to differences in outcomes. Nevertheless, Chen et al. (46), noted that varying in follow-up durations across studies impact the assessment of outcomes, which emphasized the need for standardized reporting in surgical studies to enhance comparability.

Another important consideration is the cost of robotic surgery. RAS generally incurs substantially higher operative and equipment-related costs compared with CLS due to capital investment, maintenance, and disposable instrument expenses. Although reduced conversion and complication rates may offset part of the expense, current evidence remains mixed, and cost-effectiveness is highly dependent on institutional volume and resource allocation (47). Future economic evaluations are needed to determine whether the perioperative benefits justify the additional cost.

Our study has several limitations. First, our results are based on aggregate-level data extracted from published reports, which may introduce bias arising from differences in study design, surgical techniques, and the definitions used for reporting postoperative complications. Second, the interpretation of postoperative bowel recovery outcomes—particularly time to first flatus—is limited by inconsistent reporting and variable implementation of enhanced recovery after surgery (ERAS) protocols across studies, which may independently influence gastrointestinal functional recovery. Third, the search strategy was restricted to studies published between 2018 and 2024. Although this approach improves comparability by focusing on contemporary robotic platforms and standardized perioperative care, it may have excluded older but methodologically robust trials. Finally, the absence of patient-level data prevents definitive assessment of long-term oncologic outcomes and survival differences between RAS and CLS. These limitations should be considered when interpreting the pooled results.

In the future, prospective multicentre randomized controlled studies should be performed in a larger sample size to achieve more reliable evidence of the superiority of RAS over CLS. If surgical techniques and criteria for grading of complications can be standardized, study comparison will be facilitated and a more definitive answer may then emerge. Furthermore, understanding patient-related factors affecting surgical results may contribute to individualized treatment modalities and improved clinical outcome of colorectal cancer patients.

**In conclusion**, this meta-analysis suggests that RAS may reduce the incidence of open conversion and reoperation rates, at the expense of longer duration of operative time. Our findings contribute to the growing body of literature supporting the use of robotic surgery in colorectal cancer, highlighting the need for continued exploration of its role in surgical practice.

## Data Availability

The original contributions presented in the study are included in the article/Supplementary Material, further inquiries can be directed to the corresponding author.
