# Supplementary material for: Perioperative outcomes of robotic-assisted vs. conventional laparoscopy for colorectal cancer resection: a systematic review and meta-analysis
Source: Front Surg. 2026 Feb 25;13:1723076. doi: 10.3389/fsurg.2026.1723076 (PMC12975735; doi:10.3389/fsurg.2026.1723076)
Supplement: Supplementary file 1 [file Supplementaryfile1.docx]

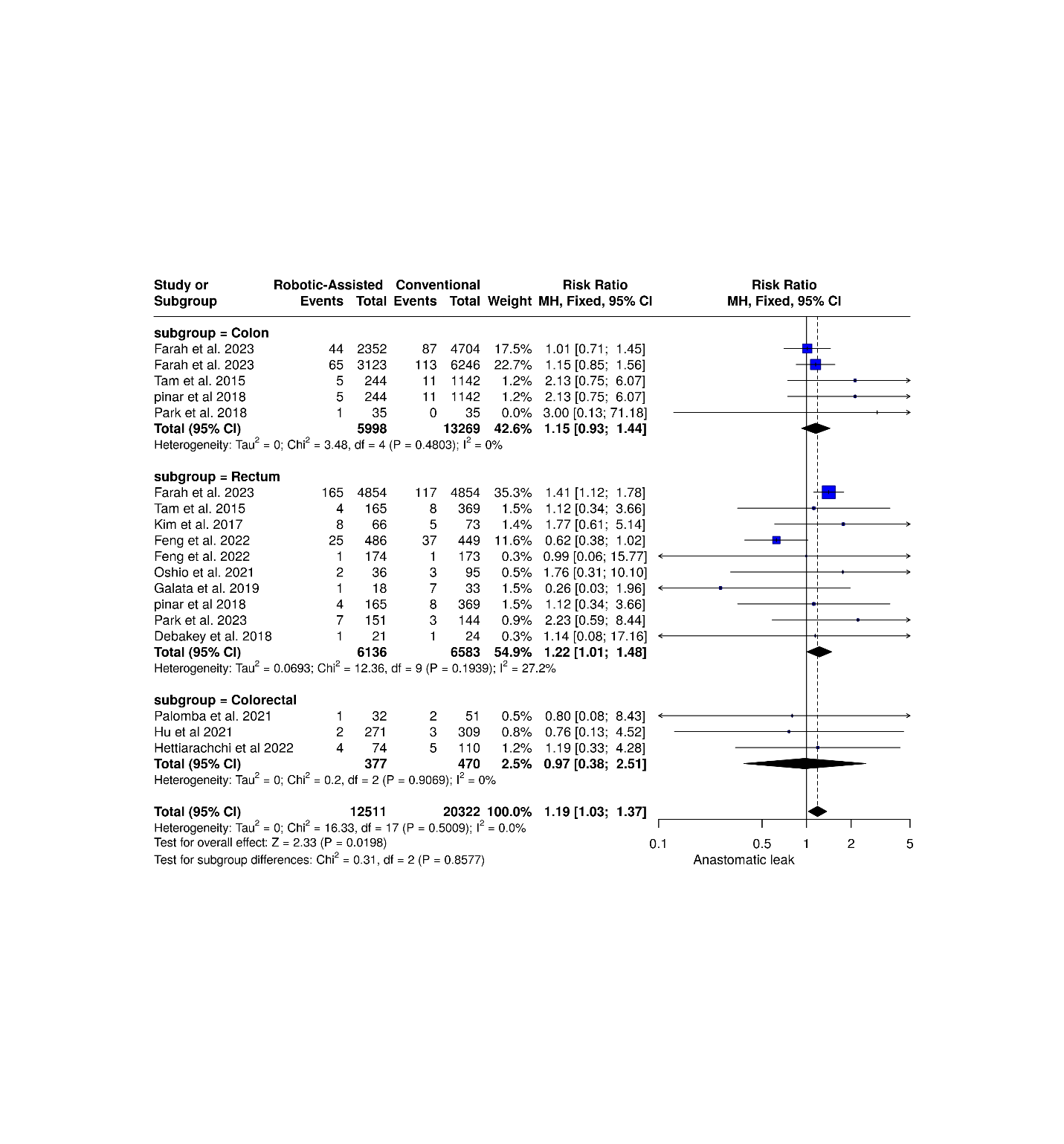


Figure 1s Forest plots illustrate the analysis of anastomatic leak


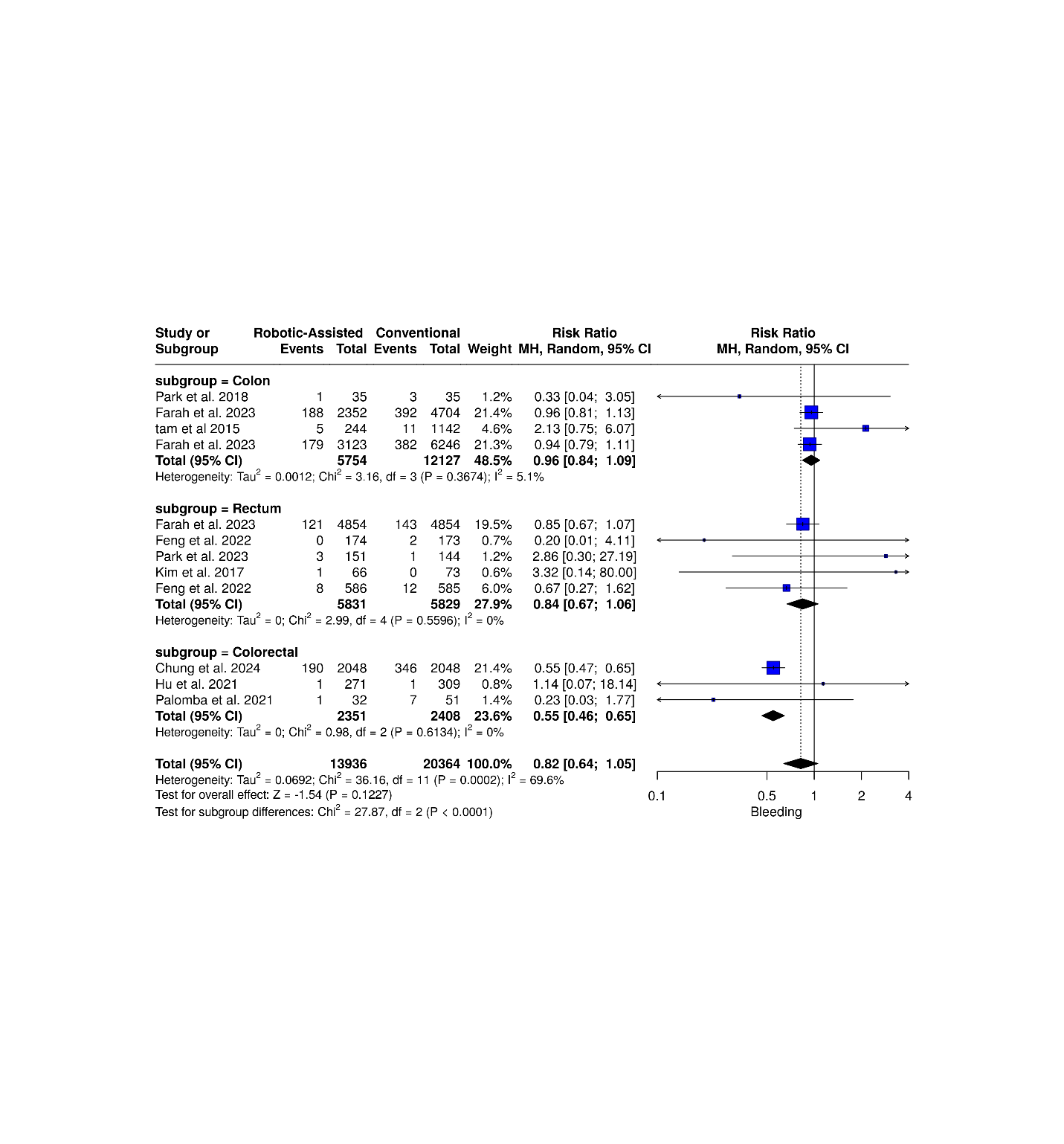


Figure 2s Forest plots illustrate the analysis of bleeding rate


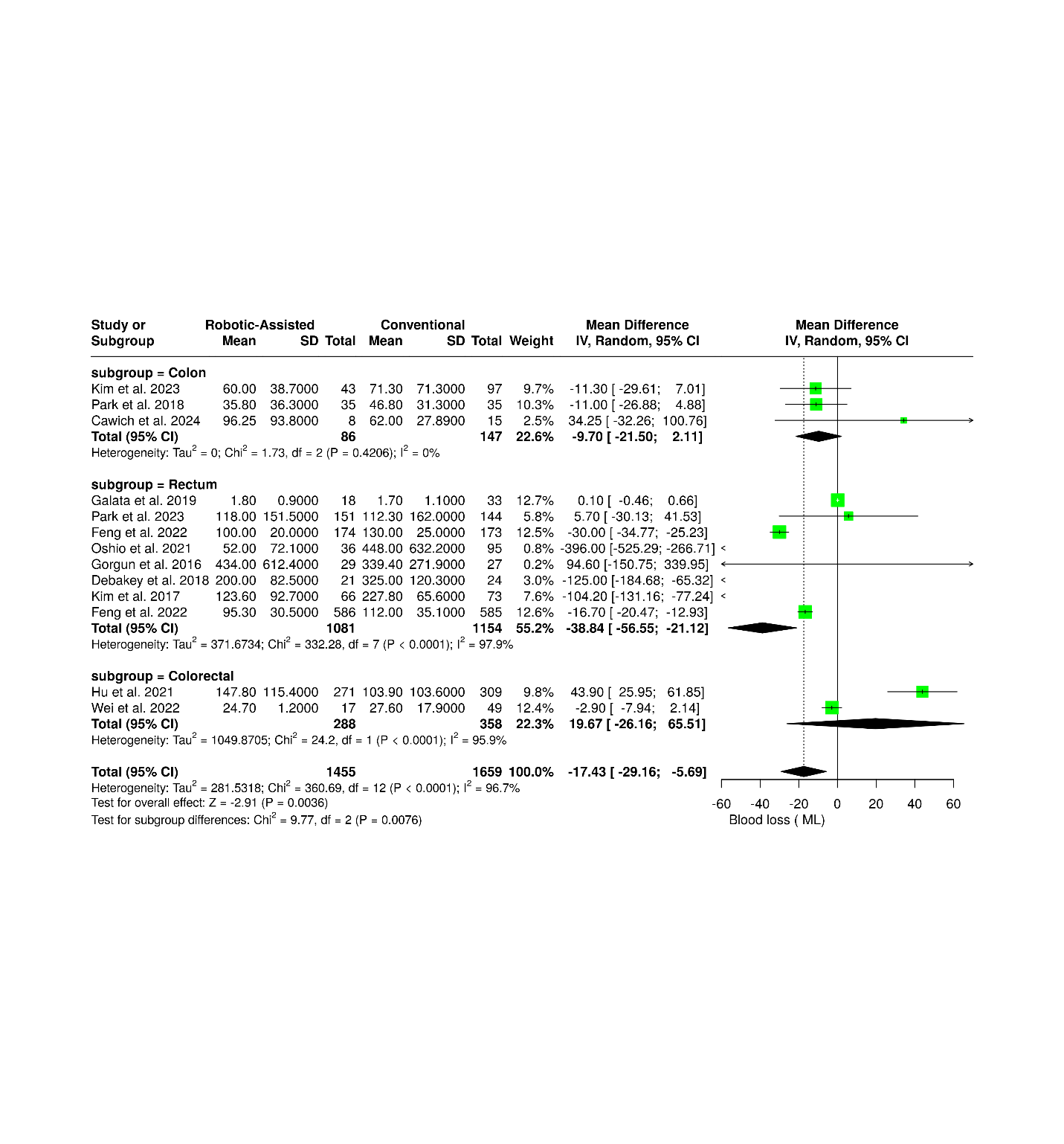


Figure 3s Forest plots illustrate the analysis of intraoperative blood loss


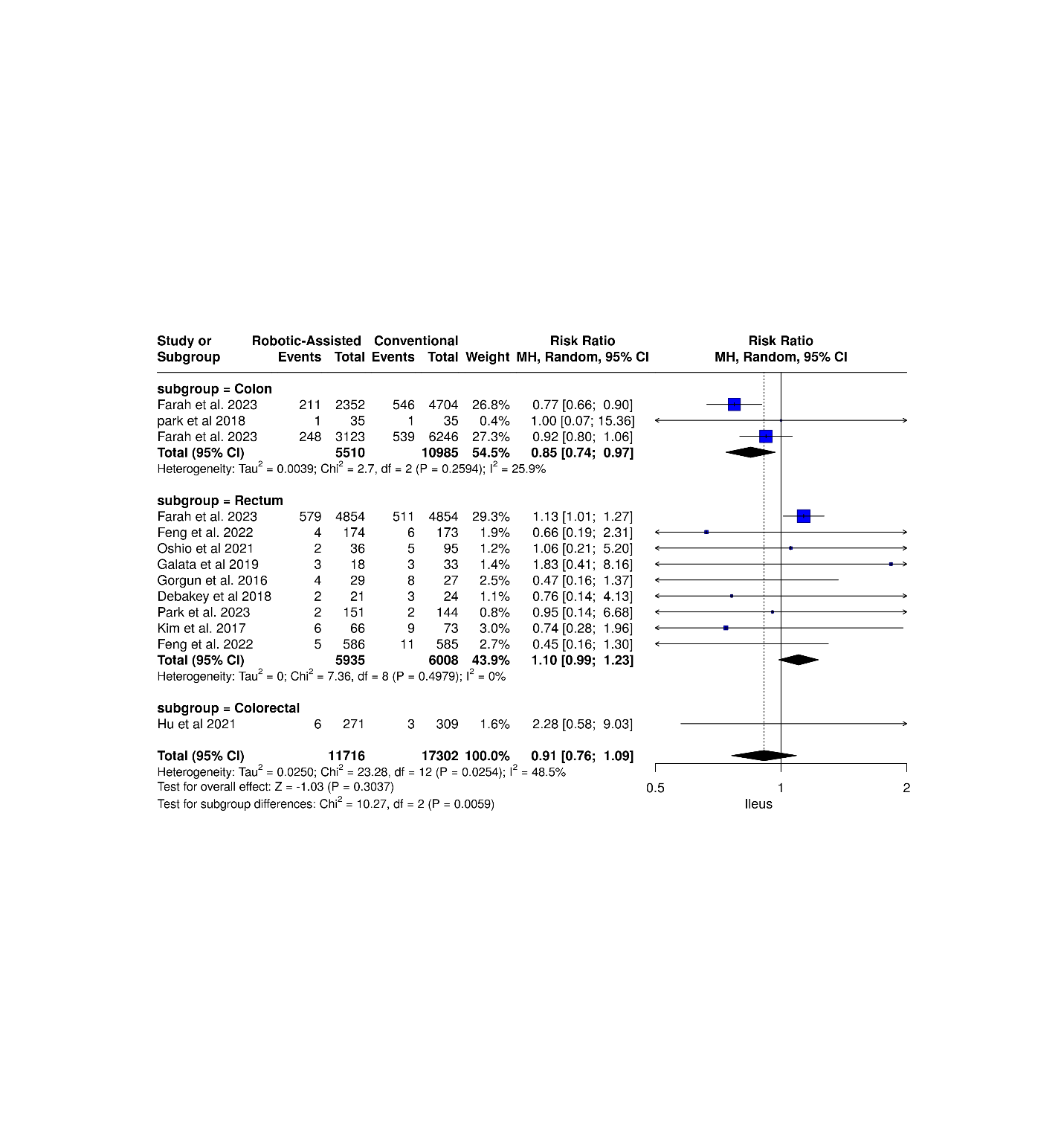


Figure 4s Forest plots illustrate the analysis of postoperative ileus


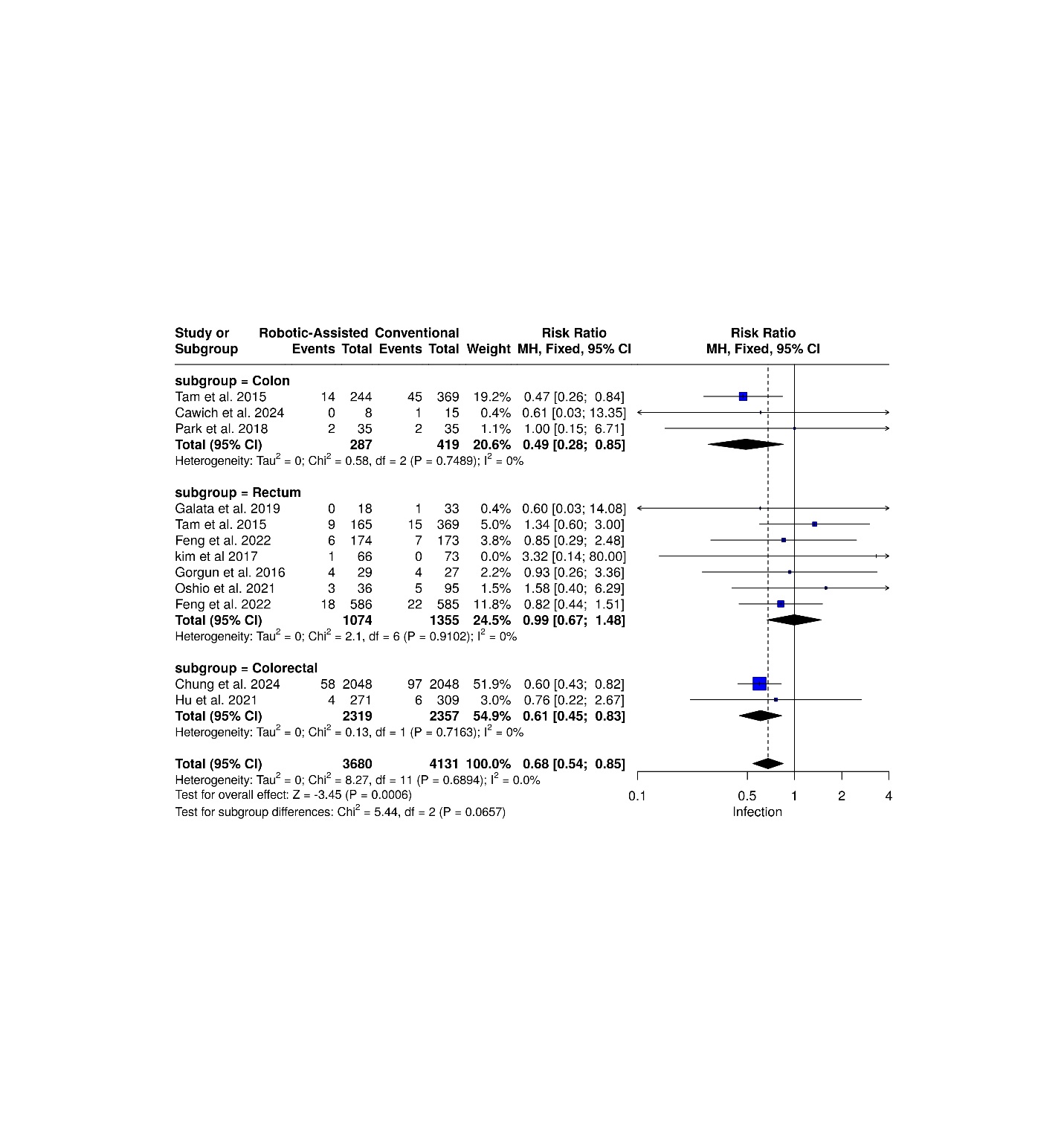


Figure 5s Forest plots illustrate the analysis of infection rate


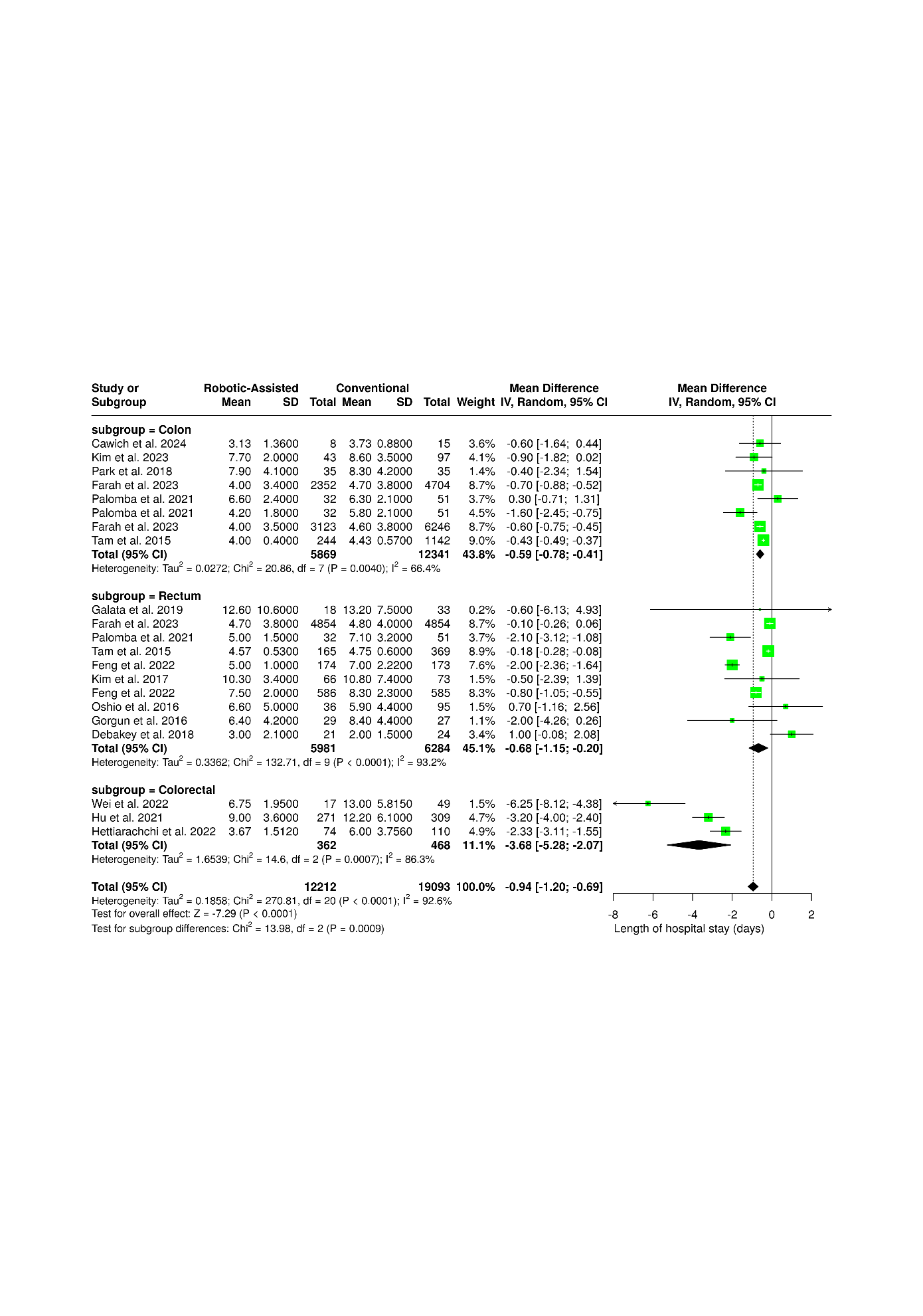


Figure 6s Forest plots illustrate the analysis of length of hospital stay


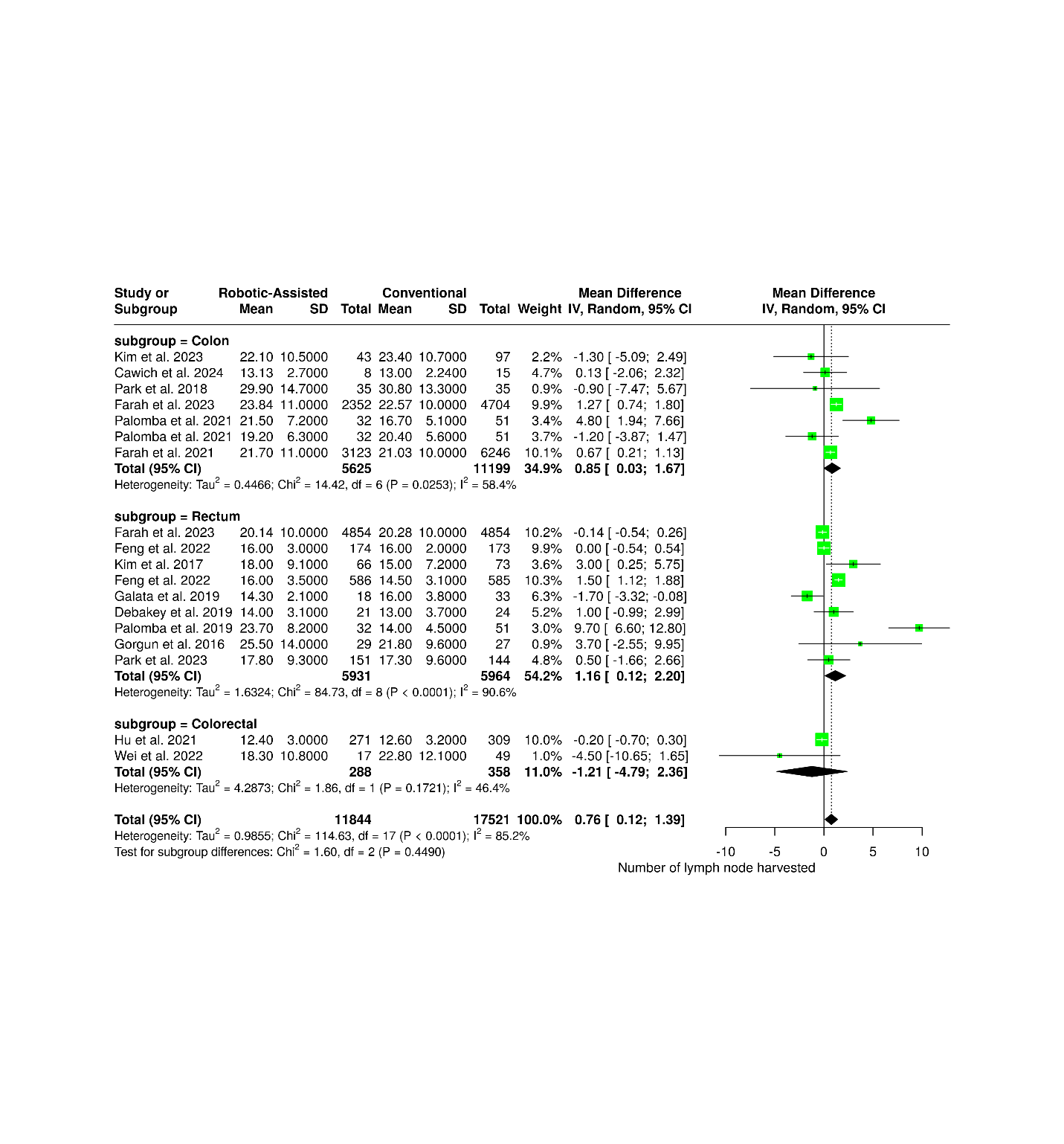


Figure 7s Forest plots illustrate the analysis of number of lymph node harvested


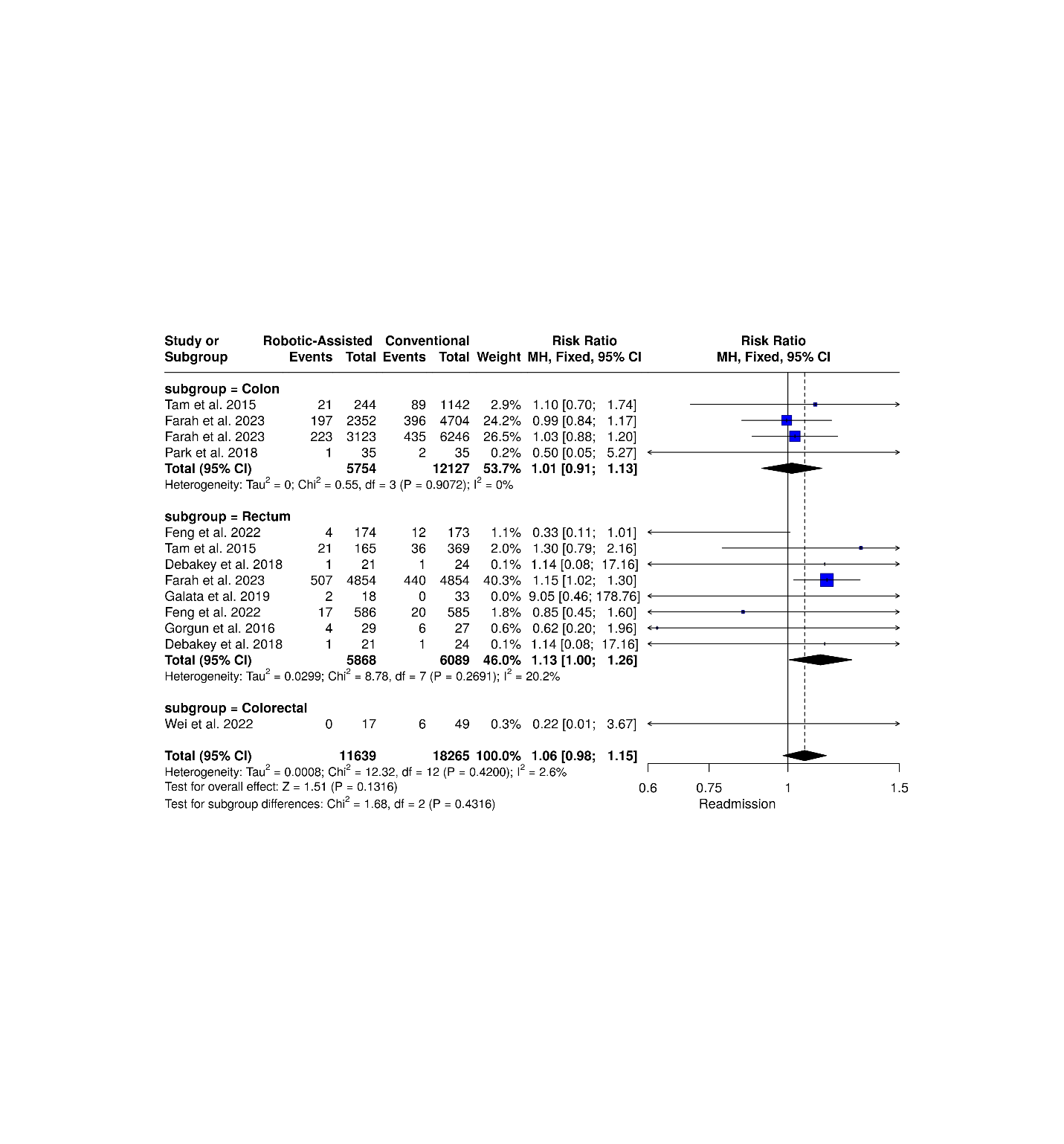


Figure 8s Forest plots illustrate the analysis of readmission rate


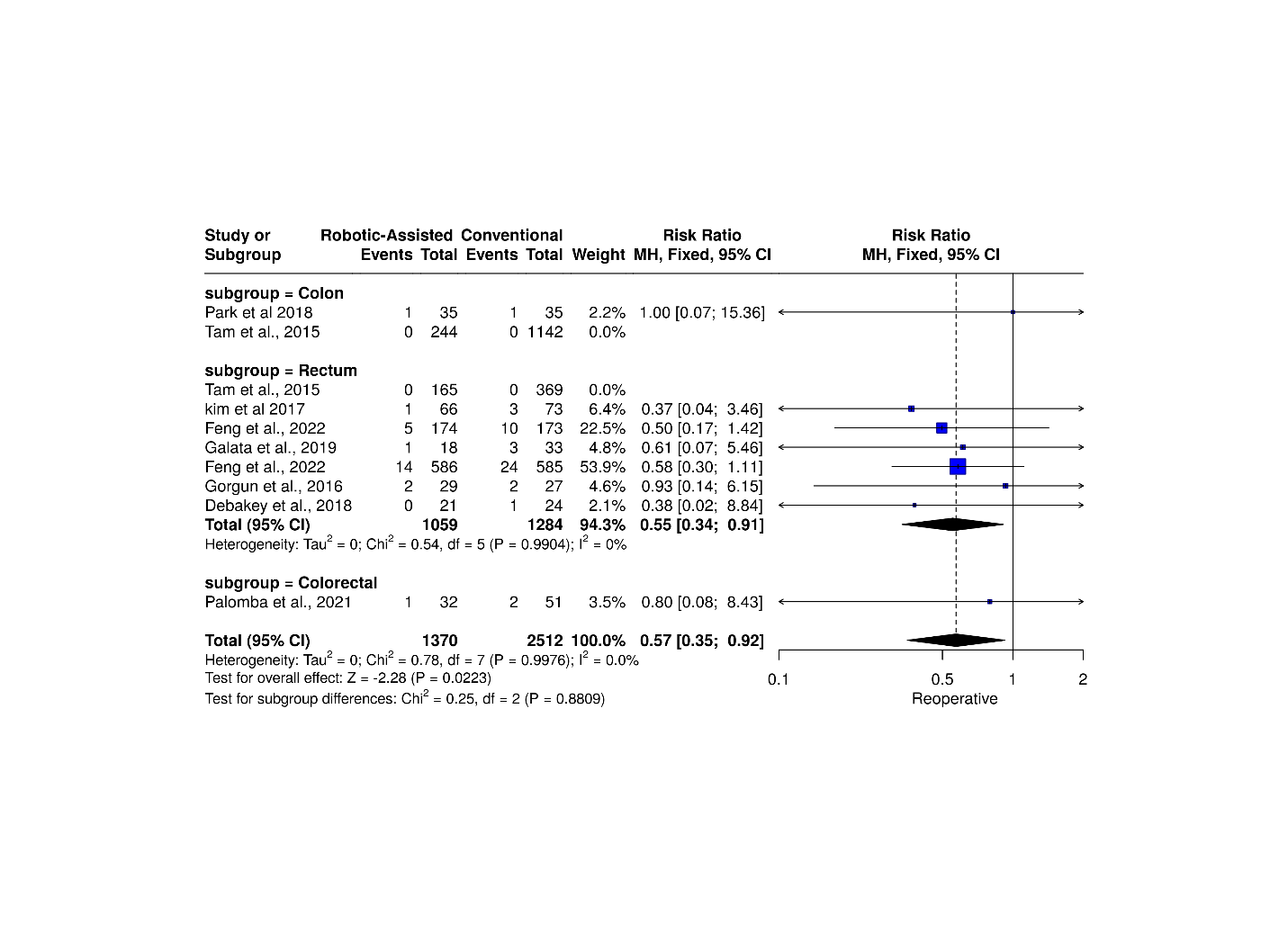


Figure 9s Forest plots illustrate the analysis of reoperative rate


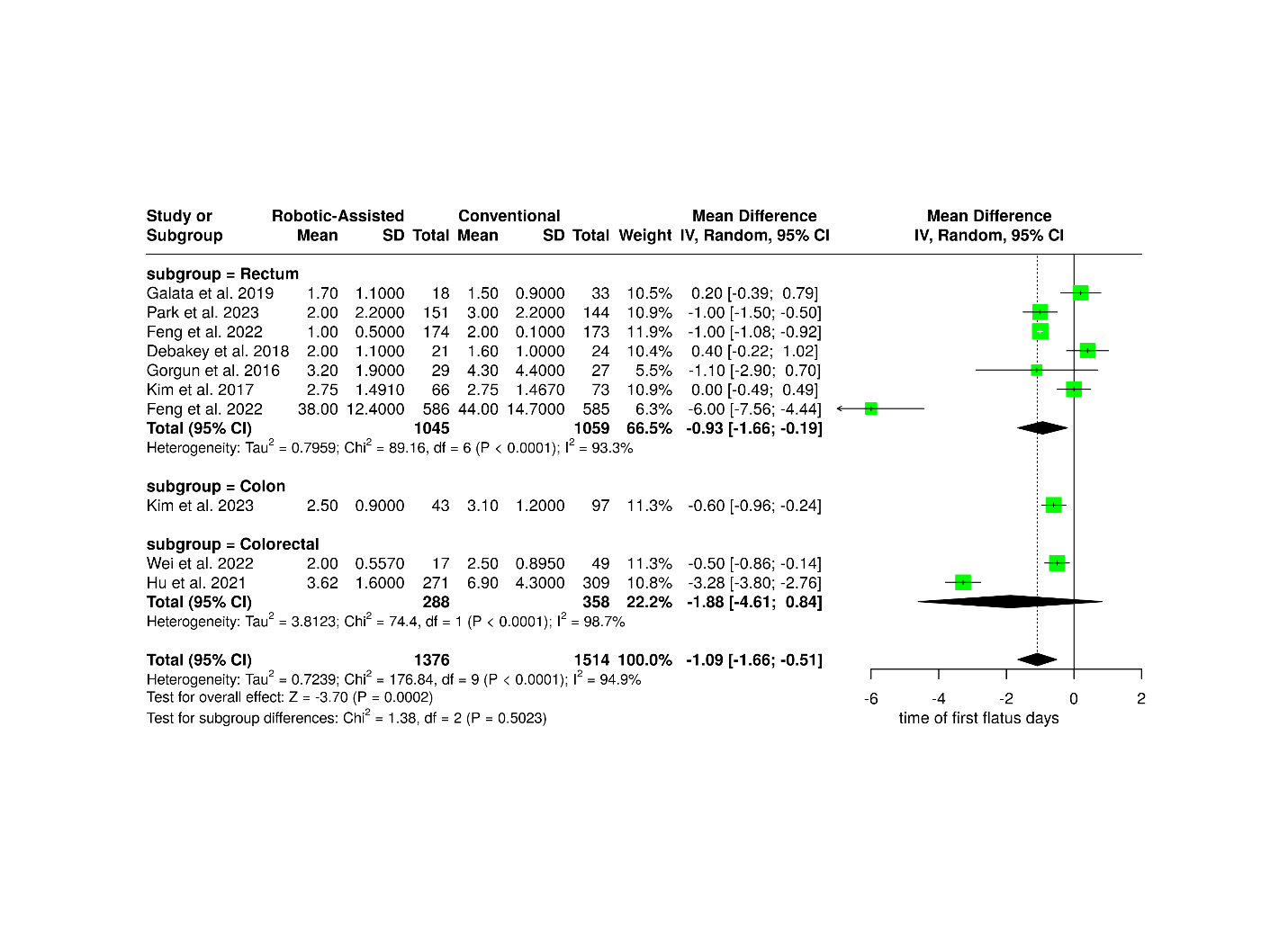


Figure 10s Forest plots illustrate the analysis of time to first flatus


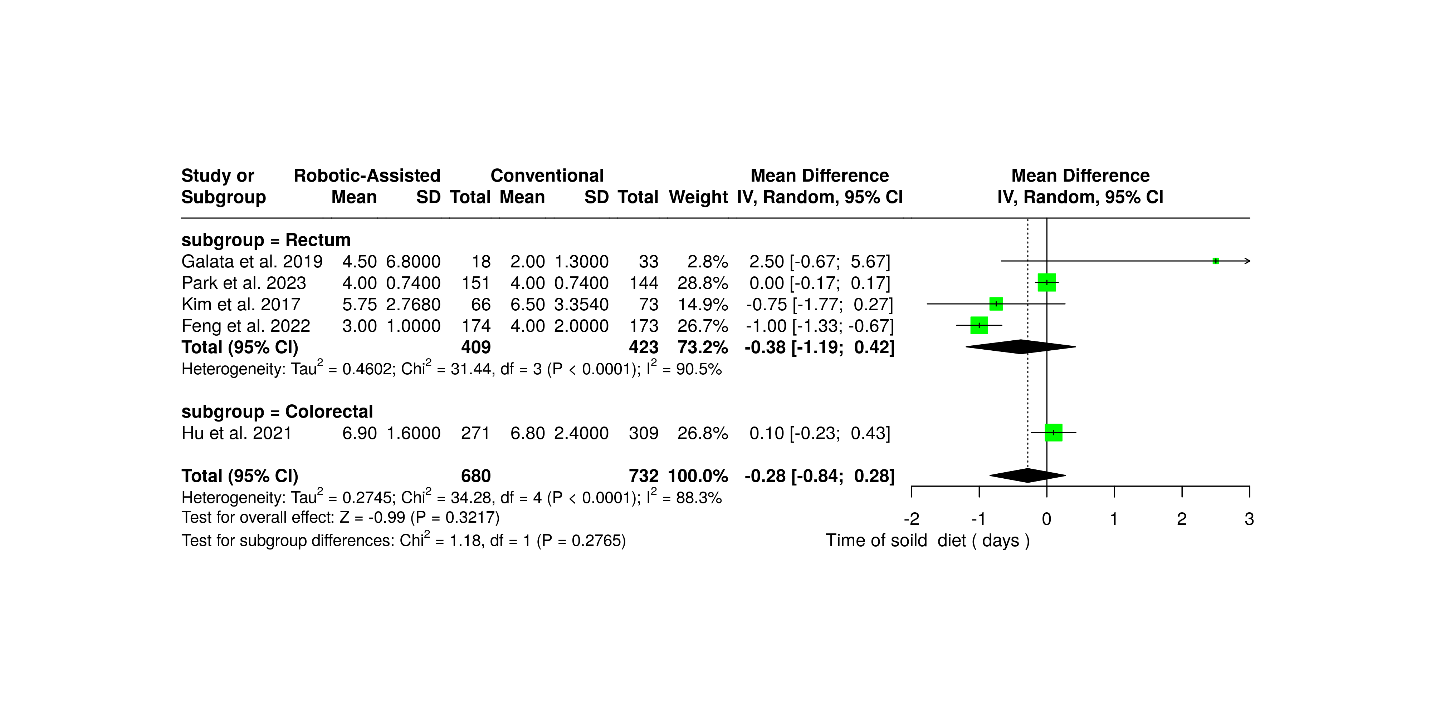


Figure 11s Forest plots illustrate the analysis of time to resume a soft diet


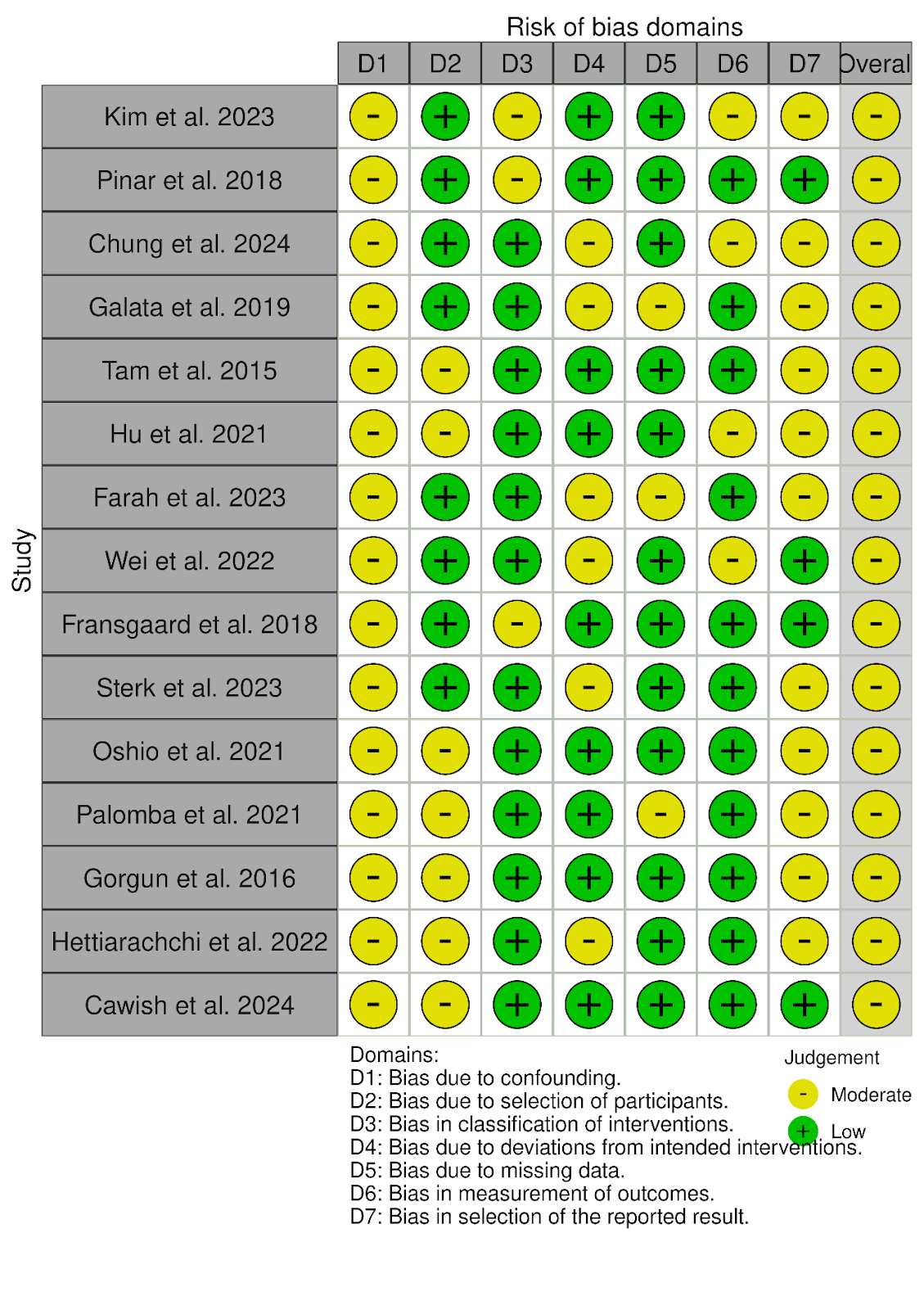


Figure 12s Risk of Bias assessment for observational studies using ROBINS-1 tool


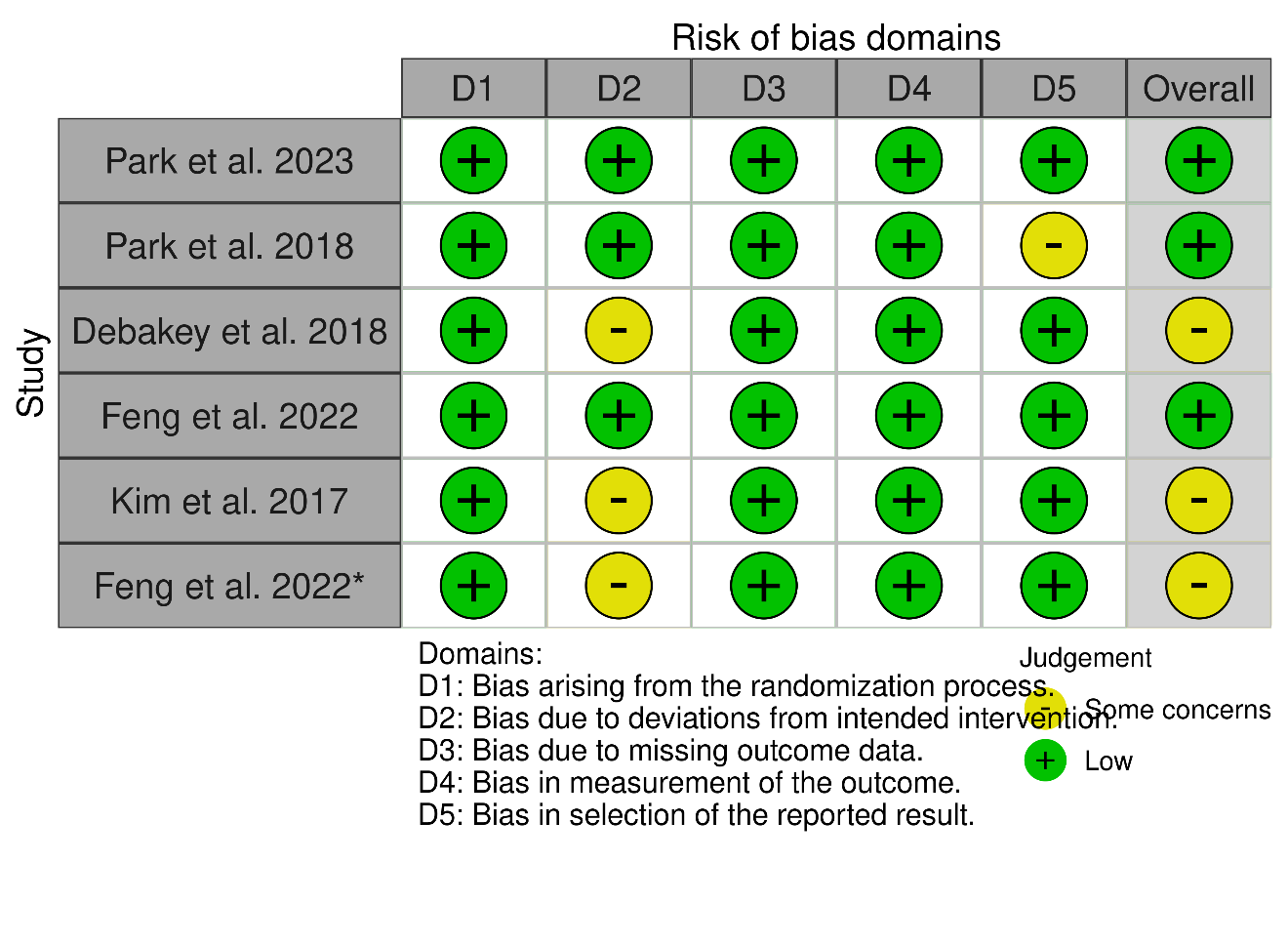


Figure 13s Risk of Bias assessment for Randomized studies using ROB-2 tool
